# Spectral CT quantification stability and accuracy for pediatric patients: A phantom study

**DOI:** 10.1002/acm2.13161

**Published:** 2021-01-10

**Authors:** Nadav Shapira, Kai Mei, Peter B. Noël

**Affiliations:** ^1^ Department of Radiology University of Pennsylvania Philadelphia USA; ^2^ Department of Diagnostic and Interventional Radiology Technical University of Munich Munich Germany

**Keywords:** dual energy CT, pediatric imagining, quantitative imaging, spectral CT

## Abstract

**Background:**

Spectral computed tomography (spectral CT) provides access to clinically relevant measures of endogenous and exogenous materials in patients. For pediatric patients, current spectral CT applications include lesion characterization, quantitative vascular imaging, assessments of tumor response to treatment, and more.

**Objective:**

The aim of this study is a comprehensive investigation of the accuracy and stability of spectral quantifications from a spectral detector‐based CT system with respect to different patient sizes and radiation dose levels relevant for the pediatric population.

**Materials and methods:**

A spectral CT phantom with tissue‐mimicking materials and iodine concentrations relevant for pediatric imaging was scanned on a spectral detector CT system using a standard pediatric abdominal protocol at 100%, 67%, 33% and 10% of the nominal radiation dose level. Different pediatric patient sizes were simulated using supplemental 3D‐printed extension rings. Virtual mono‐energetic, iodine density, effective atomic number, and electron density results were analyzed for stability with respect to radiation dose and patient size.

**Results:**

Compared to conventional CT imaging, a pronounced improvement in the stability of attenuation measurements across patient size was observed when using virtual mono‐energetic images. Iodine densities were within 0.1 mg/ml, effective atomic numbers were within 0.26 atomic numbers and electron density quantifications were within ±1.0% of their respective nominal values. Relative to the nominal dose clinical protocol, differences in attenuation of all tissue‐mimicking materials were maintained below 1.6 HU for a 33% dose reduction, below 2.7 HU for a 67% dose reduction and below 3.7 HU for a 90% dose reduction, for all virtual mono‐energetic energies equal to or greater than 50 keV. Iodine, and effective atomic number quantifications were stable to within 0.1 mg/ml and 0.06 atomic numbers, respectively, across all measured dose levels.

**Conclusion:**

Spectral CT provides accurate and stable material quantification with respect to radiation dose reduction (up to 90%) and differing pediatric patient size. The observed consistency is an important step towards quantitative pediatric imaging at low radiation exposure levels.

## 1 INTRODUCTION

Spectral computed tomography (spectral CT) provides quantitative information that enhances conspicuity of disease and tissue characterization capabilities. The quantitative capabilities of spectral CT arise from access to elemental composition information. This is achieved through measurements of the energy‐dependent material‐specific x‐ray attenuation at different energies during a single CT scan.^1^

Dual‐Energy Computed Tomography (DECT), the first realization of spectral CT, has been investigated since the 1970s and became a commercially available clinical tool more than a decade ago.^2^ DECT provides a variety of spectral results enabling quantitative measurement of endogenous and exogenous materials within the patient. Spectral results include virtual mono‐energetic images that, unlike conventional CT images, provide well‐defined attenuation quantifications that enhance conspicuity and tissue characterization capabilities^3^, ^4^ while reducing metal and beam‐hardening artifacts.^3^, ^5^ Other spectral results provide material‐specific quantifications that enable density assessment and virtual attenuation suppression of various clinically relevant materials, e.g. iodine^6^, ^7^ and calcium^8^ or electron density (ED) or effective atomic number (Zeff) estimations that enable improved material differentiation.^6^ Recommended applications of spectral CT for children currently include vascular imaging, bowel imaging, assessment of perfused lung blood volume, detection and characterization of lesions in solid organs, tumor treatment response evaluations, metal artifact reduction, and renal calculi composition assessments.^9^ All these applications rely on accurate attenuation or material quantifications, and their clinical benefit improves with increased consistency. For example, accurate iodine maps are used to generate perfused blood volume images that map iodine distributions in the lung parenchyma and allow both visualization and quantification of perfusion defects, and energy‐dependent x‐ray attenuation profiles are utilized for classifying different types of common stones that are found in children based on their predominant materials.

Different technologies can be used to implement the concept of acquiring paired attenuation measurements from two different energy spectra. Current commercial approaches to spectral CT include the rapid kVp‐switching method,^10^ the dual‐source method,^2^ the split‐beam method,^11^ the spin‐spin method^1^ and the dual‐layer detector method,^12^ which we utilized in our study. While source‐based approaches achieve spectral separation by variations in the radiation spectra that pass through the patient, implemented either by changing the accelerating tube voltage or by different x‐ray filtrations, the only commercially available detector‐based spectral CT system owes its spectral separation capabilities to a dedicated dual‐layered detector. The unique detector design consists of a horizontal configuration of an Yttrium‐based top layer that is more sensitive to low‐energy photons and serves as filtration for the Gadolinium oxysulfide‐based bottom layer, which mostly detects high‐energy photons. Spectral information with sufficient energy separation is acquired with perfect spatial and temporal alignment at every 120 or 140 kVp scan, obviating the need to prospectively modify scan parameters in order to enable a spectral CT acquisition mode.^13^

While the proven value of spectral CT is continuously rising,^14^ there are only a few studies focused on spectral CT applications for pediatric imaging.^9^, ^15^, ^16^ For this unique population, imaging applications concentrated on a different domain of clinical questions compared to those of adult patients is of interest. In addition, research of different technical aspects that are related to image quality are of importance, specifically, with respect to the smaller sizes of pediatric patients, and, perhaps more importantly, their sensitivity to ionizing radiation and the obligation to use lower doses.^17^ Any application of CT for pediatric patients must include a risk‐benefit analysis weighing the risk of exposure to ionizing radiation with the benefit of the information obtained from the scan. This is because young children are more sensitive to the effects of radiation than adults.^18^, ^19^, ^20^

There are very few studies that include phantom measurements dedicated for pediatric patient spectral CT imaging.^21^ Of these, the ability to perform dose‐neutral spectral CT imaging was the primary concern,^22^, ^23^ rather than the resulting spectral accuracy. Comprehensive studies that evaluate the quantification capabilities of spectral CT while focusing on pediatric imaging are therefore still necessary for the adoption of spectral applications for this unique population. The aim of this study is to conduct a thorough investigation of the accuracy and stability of spectral CT quantitative measurements with respect to different patient sizes and radiation dose levels that are relevant to pediatric patients.

## 2 MATERIALS AND METHODS

### 2.A Phantoms

**FIG. 1:**
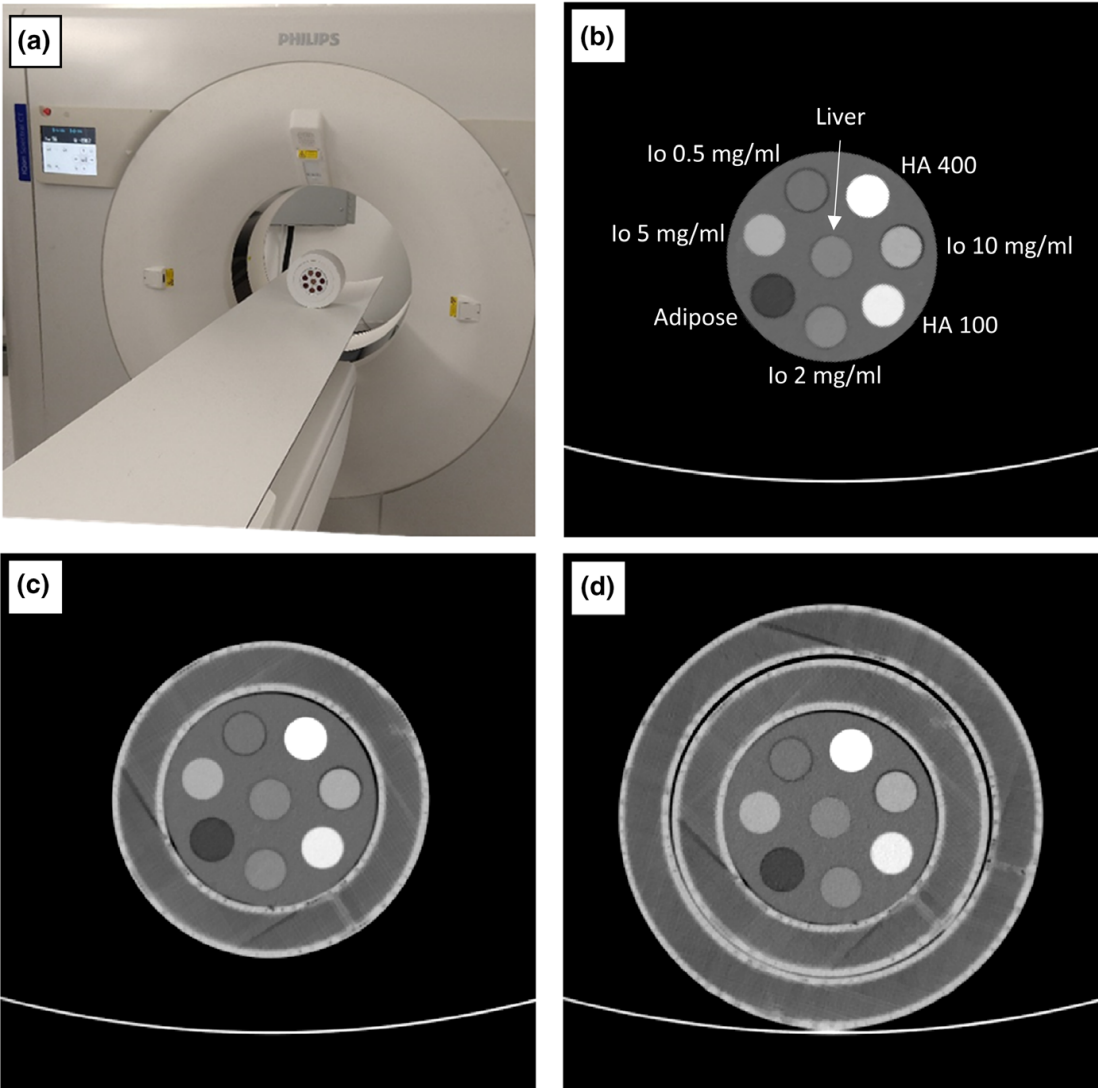
70 keV virtual mono‐energetic images results of the spectral CT pediatric phantom used to evaluate spectral accuracy as a function of patient size and radiation dose. (a) Photograph of the scanner and phantom, with both 15 and 20 cm extension rings, which were used in our study. The 10 cm diameter insert (b) contains eight different tissue‐mimicking and iodine density rods. The insert was scanned within two extension rings that have outer diameters of 15 cm (c) and 20 cm (d) to evaluate the size dependency of various spectral results. Window Level/Window Width = 50/500.

In order to assess the accuracy of various spectral results as a function of patient size and radiation dose, a custom spectral CT phantom was used (Tissue Equivalent Materials & CTIodine®, QRM GmbH, Moehrendorf, Germany). The phantom, shown in Fig. 1, contains a 10 cm diameter insert that can hold up to eight different tissue‐mimicking or iodine density rods at a time. The insert and 1 cm diameter material rods are all 10 cm in length. The insert is made of a solid water equivalent plastic with X‐ray attenuation properties similar to those of (liquid) water. Due to the importance of iodine in spectral CT imaging, we included four iodine rods at different concentrations: 0.5, 2, 5, and 10 mg/ml. The remaining rods were made of liver, adipose, 100 mg/ml hydroxyapatite (HA) and 400 mg/ml HA tissue‐mimicking materials (HA simulates bone).

Reported waist circumference measurements in infants and children^24^, ^25^ were converted into approximate body diameters (through division by π) to determine median patient sizes of newborns, 1.5‐2 yr‐old, and 9‐yr‐old patients and select the phantom sizes (10–20 cm diameter) utilized in this study. That said, the large variability in waist circumferences at these early ages imply that, for example, a 20 cm diameter phantom size corresponds to the 85th percentile of 5‐yr‐old female patients as well as the 10th percentile of 13‐yr‐old male patients such that imaging of the selected phantom sizes are relevant for some older patients. In order to evaluate the size dependency of various spectral results, the insert was scanned at three different configurations: alone, within a single 3D‐printed extension ring, and within two 3D‐printed extension rings [Figs. 1(b), 1(c)]. The extension rings, 10 cm in length and 15 or 20 cm in diameter, were fabricated from a PLA (polylactate acid) filament by using a 3D printer (F400 printer by Fusion3, Greensboro, NC, USA) with a 0.4 mm nozzle. The infill setting was set to 80%, with densely rendered outer shells. The attenuation of the interior of the rings varied between −5 and −25 HU, as measured on a conventional 120 kVp CT image, providing a reasonable approximation for a fat and soft tissue mixture. Polymeric foam layers of different thicknesses raise the insert and the 15 cm extension ring in order to achieve similar positioning of the material rods between the different phantom configurations (Fig. 1).

### 2.B Scanner and acquisition protocol

The experiments were performed on a commercially available spectral detector (dual‐layer) CT system (IQon Spectral CT, Philips Healthcare, Eindhoven, The Netherlands). The system is based on a conventional multi‐detector CT system with rotation times down to 0.27 s, a 700 mm bore and a 120 kW generator allowing tube voltages of 80, 100, 120, and 140 kVp. The system is equipped with a 4 cm dual‐layer spectral detector where the top layer is more sensitive to low‐energy photons and serves as filtration for the bottom layer, which mostly detects high‐energy photons. Detector materials and thicknesses were designed to result in equal noise levels for scans of typical body sizes. Spectral information with sufficient energy separation is acquired at every 120 or 140 kVp scan, eliminating the need to prospectively modify scan parameters in order to enable a spectral CT acquisition mode.^13^ Owing to the detector‐based spectral separation design of the system, data are acquired with perfectly aligned spatial and temporal registration of the two spectra in the projection domain. This provides the opportunity to accurately account for beam‐hardening effects and to achieve highly efficient noise reduction with dedicated projection‐based material decomposition and spectral denoising algorithms.^12^, ^26^, ^27^ Several spectral results are available both prospectively and retrospectively. These include Virtual Mono‐energetic Images (VMI) at photon energy levels of 40 to 200 keV, iodine density maps, Virtual Noncontrast (VNC) images, effective atomic number images and electron density images.

The phantom was positioned in the iso‐center. All scans were performed using the nominal pediatric (0–10 kg) abdomen scan protocol, where the only modified parameters were the radiation dose level, and the spectral denoising level. The protocol utilizes a tube voltage of 120 kVp, a 4 cm collimation (64×0.625 mm), a rotation time of 0.27 s, a pitch factor of 1.234, and a mid‐range spectral denoising level of 4. In order to study the stability of the various spectral results on patient dose, three repetitive acquisitions were performed for each of three phantom configurations at the four following tube current‐time product values: 100 mAs (nominal, 100%), 67, 33 and 10 mAs. These correspond to CTDI_vol_ dose levels of 9, 6, 3, and 0.9 mGy, or effective dose levels of 0.18, 0.12, 0.06, and 0.018 mSv per every 10 cm scanned, when using the appropriate conversion (k) factor of 0.02 for abdomen and pelvis of 5‐yr‐old pediatric patients.^28^

Reconstruction of each acquisition was performed using the clinical standard body kernel (“B” on the IQon scanner), a reconstruction field of view (FOV) diameter of 250 mm, 3 mm slice thicknesses and increments, and a matrix size of 512 × 512 pixels. Each reconstruction resulted in a series of spectral base images (SBI) which contain all the information that is required to generate all available spectral results. From these SBIs, VMIs of various energies as well as iodine density, Zeff, and ED images were generated.

### 2.C Nominal values calculations

The nominal attenuation at various VMI energies [HU(E)] for the four tissue‐mimicking rods were calculated based on the material composition (given as normalized elemental mass fractions, $w_{i}$) and physical density ($\rho_{\mathrm{phys}}$) of each rod, which were provided by the phantom manufacturer, according to:

(1) $$HU\left(E\right)=1000\times \left(\frac{\rho_{\mathrm{phys}}\sum_{i}w_{i}\times \mu_{i}\left(E\right)}{\mu_{\mathrm{water}}\left(E\right)}‐1\right)$$

where $\mu_{i}\left(E\right)$ and $\mu_{\mathrm{water}}\left(E\right)$ are the mass attenuation coefficients (expressed in cm^2^/g) at photon energy Eof elementi and water, respectively, and the sum is over all elements in the elemental composition. Values for $\mu_{i}\left(E\right)$ of the various elements within the elemental compositions of the rods and for water were obtained from publicly available data provided by the National Institute of Standards and Technology (NIST).^29^

Similarly, nominal effective atomic number (Zeff) values for the four tissue‐mimicking rods were calculated according to:

(2) $$Z_{\mathrm{eff}}=\sqrt[{n_{Z}}]{\sum_{i}f_{i}Z_{i}^{n_{Z}}}$$

where $Z_{i}$ is the atomic number of each element in the rod composition, $f_{i}=\left(w_{i}Z_{i}/A_{i}\right)/\sum_{i}\left(w_{i}Z_{i}/A_{i}\right)$ is the fraction of the total number of electrons associated with each element, and $A_{i}$ is the atomic mass of each element.^6^, ^30^ While the general form of Eq. 2 is widely accepted, different values of $n_{Z}$ appear in different publications.^31^, ^32^ For our analysis, we have chosen to use $n_{z}=2.94$ since this is the value that was adopted by Philips for generating this result.^6^

Finally, the nominal electron density (ED) values were calculated according to:

(3) $$ED=100\times \frac{N_{A}\rho_{\mathrm{phys}}}{ED_{\mathrm{water}}}\sum_{i}\frac{w_{i}}{A_{i}}Z_{i}$$

where $ED_{\mathrm{water}}$ is the electron density of water ($3.343\times 10^{23}m^{‐3}$) and $N_{A}=6.02214\times 10^{23}mol^{‐1}$ is Avogadro’s number. The ED values provided by Eq. (3) are normalized to water and converted to percent, i.e., %ED_water_, since these are the units of the ED spectral result on the IQon scanner. The calculated HU, Zeff and ED values for the tissue‐mimicking rods is provided in Table 1, together with errors which arise from uncertainties in physical densities $\rho_{\mathrm{phys}}$, which according to the phantom manufacturer are ±0.02 mg/ml for all material rods.

**TABLE 1:** Nominal values for the evaluated virtual mono‐energetic images (VMI), electron density and effective atomic number (Zeff) results in the study, together with mean measurement values achieved with the highest radiation dose and reference patient size of 15 cm. Possible errors in the nominal values arise from uncertainties in material densities (0.02 mg/ml according to the phantom manufacturer).

Tisssue‐mimicking rods		Liver	Adipose	HA‐100	HA‐400
VMI 40 keV [HU]	Expected	58.7 ± 19.9	−155.5 ± 17.4	311.4 ± 24.1	1324.8 ± 36.1
	Difference	15.9 ± 4.9	11.4 ± 2.7	3.4 ± 4.0	18.1 ± 12.5
VMI 50 keV [HU]	Expected	52.6 ± 19.8	−112.0 ± 18.3	212.2 ± 22.3	893.6 ± 29.4
	Difference	14.1 ± 3.2	7.3 ± 1.8	10.1 ± 2.6	13.0 ± 7.9
VMI 60 keV [HU]	Expected	48.8 ± 19.7	−88.2 ± 18.8	156.9 ± 21.3	654.3 ± 25.7
	Difference	13.2 ± 2.3	6.7 ± 1.3	10.9 ± 2.0	17.1 ± 5.2
VMI 67 keV [HU]	Expected	46.8 ± 19.7	−78.1 ± 19.0	132.5 ± 20.8	549.6 ± 24.1
	Difference	13.2 ± 1.9	7.4 ± 1.1	11.1 ± 1.6	16.0 ± 4.0
VMI 70 keV [HU]	Expected	46.3 ± 19.7	−74.7 ± 19.0	124.6 ± 20.6	515.4 ± 23.5
	Difference	13.0 ± 1.8	7.2 ± 1.0	11.0 ± 1.6	15.2 ± 3.6
VMI 100 keV [HU]	Expected	43.8 ± 19.6	−57.3 ± 19.5	84.6 ± 20.0	342.1 ± 20.8
	Difference	11.7 ± 1.3	8.2 ± 0.9	7.7 ± 1.4	2.6 ± 1.8
VMI 150 keV [HU]	Expected	42.7 ± 19.5	−50.0 ± 19.6	67.9 ± 19.6	269.2 ± 19.7
	Difference	11.1 ± 1.2	9.0 ± 1.0	5.4 ± 1.4	7.5 ± 1.5
VMI 200 keV [HU]	Expected	42.4 ± 19.6	−47.9 ± 19.6	63.4 ± 19.5	249.3 ± 19.4
	Difference	11.0 ± 1.1	9.6 ± 1.0	4.2 ± 1.3	12.0 ± 1.5
ED [%ED_water_]	Expected	104.3 ± 2.0	95.5 ± 2.0	106.0 ± 1.9	123.0 ± 1.9
	Difference	1.0 ± 0.1	0.8 ± 0.1	0.5 ± 0.1	0.7 ± 0.2
Zeff [Z]	Expected	7.32	6.28	8.64	11.17
	Difference	0.10 ± 0.04	0.26 ± 0.04	0.15 ± 0.02	0.21 ± 0.03

Iodine rods		0.5 mg/ml	2 mg/ml	5 mg/ml	10 mg/ml
Iodine Density [mg/ml]	Expected	0.50 ± 0.01	2.00 ± 0.04	5.00 ± 0.10	10.00 ± 0.20
	Difference	0.06 ± 0.04	0.10 ± 0.07	0.02 ± 0.05	0.08 ± 0.11

### 2.D Evaluation matrices

Analysis was based on mean and standard deviation measurements from circular regions‐of‐interest (ROIs). Eight ROIs were placed on 26 consecutive center slices from each of the 36 acquisitions (3 phantom configurations x 4 dose levels x 3 repetitions), resulting in a total of ~7500 ROIs for each spectral result. For each of the phantom configurations, rod centers ($x_{0},y_{0}$) were manually drawn on the 70 keV VMI result of one of the 100 mAs dose acquisitions and copied to all of the slices that correspond to the specific phantom configuration. All voxels that satisfy $\sqrt{{\left(x‐x_{0}\right)}^{2}+{\left(y‐y_{o}\right)}^{2}}\leq R$ with R=5mm×FOV/matrixsize≈2.5pixel (50% of the rod radius, 236 mm^3^) were averaged and their standard deviation was computed.

Analysis of phantom size dependency and radiation dose dependency was performed with the use of error bar plots and Bland‐Altman 95% “limits of agreement” analysis^33^ for the measured mean quantification values. In both plot types, errors represent a single standard deviation in each direction. In addition, maximal absolute differences and standard deviations of mean values between different phantom configurations were evaluated in order to quantify spectral assessment consistency between different patient sizes. HU were converted to CT numbers (with *air* as a reference point) in order to evaluate relative deviations in percent for the different VMI results. Finally, differences of mean values and their standard deviation relative to values at 100 mAs, were calculated for acquisitions with lower than nominal tube currents in order to assess the effect of lower patient doses on spectral accuracy.

## 3 RESULTS

The focus of this study is the stability of available spectral results with respect to radiation exposure and patient size. In order to benchmark the accuracy of the various spectral results, we have chosen to use scans of the middle phantom size (15 cm) that were acquired with the highest radiation exposure level tested in our experiment, i.e., with a nominal tube current of 100 mAs that corresponds to 9 mGy in dose, as a reference point. For this phantom size and dose configuration, iodine density results were 0.56 ± 0.04, 2.10 ± 0.07, 5.02 ± 0.05, and 10.08 ± 0.11 mg/ml, compared to the nominal values of 0.5, 2, 5 and 10 mg/ml. The accuracies of the rest of the measured spectral results that were evaluated in this study are presented in Table 1.

**FIG. 2:**
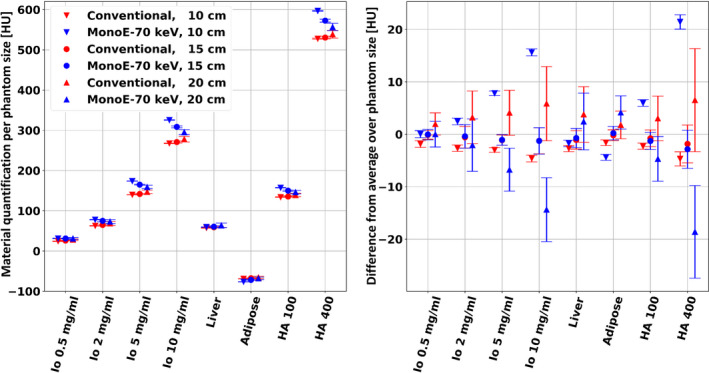
Conventional CT and 70 keV virtual mono‐energetic images (VMI) measured mean HU values of the eight material rods as a function of phantom/patient size. Scans were performed with the nominal pediatric abdomen protocol (100 mAs). Left: quantification results, in [HU], averaged over 78 slices from three repetitive scans for each phantom/patient size. Right: differences in the quantification results from the size‐averaged HU value calculated per each material rod and per each result. A significantly increased size stability (independence) is observed for the 70 keV VMI result, as compared to the conventional result.

Figure 2(a) presents measured mean HU of the conventional images and the 70 keV VMI spectral results from scans of the three different phantom configurations that were acquired with 100 mAs. About 70 keV is the default energy level of the VMI spectral result for the nominal pediatric (0–10 kg) abdomen protocol on the scanner. Changes in quantification due to patient size dependence are visualized as vertical trends within the measurements of each material. These trends are emphasized in Fig. 2(b), where each measurement is presented relative to the value of the average quantification of each material (i.e., average material‐specific quantifications per result were subtracted from the measurements). Except for the liver and 0.5 mg/ml iodine rod, the conventional results exhibit higher absolute differences between different phantom sizes. This is most significant for the HA‐400 tissue‐mimicking rod, where the absolute difference of the conventional result is 40 HU while that of the 70 keV VMI spectral result is 11.2 HU, almost a fourfold improvement in consistency. Bland‐Altman analysis of these data reveal 95% “limits of agreement” of ‐2.06 ± 0.69 HU (10 cm vs 15 cm patient size) and −4.65 ± 2.25 HU (15 cm vs 20 cm patient size) for the 70 keV VMI spectral result, compared to 6.82 ± 9.73 HU (10 cm vs 15 cm patient size) and 4.06 ± 7.17 HU (15 cm vs 20 cm patient size) for the conventional images.

**FIG. 3:**
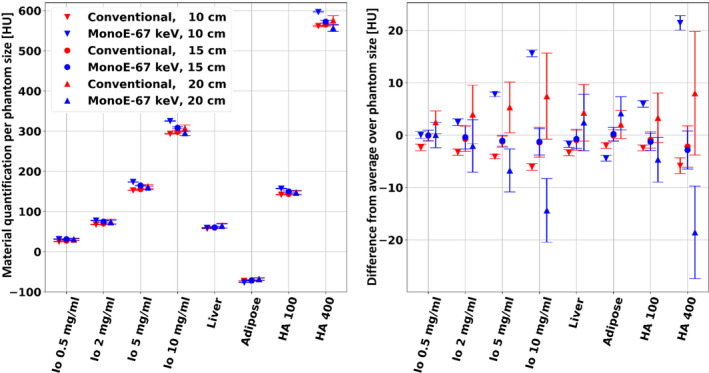
Conventional CT and 67 keV virtual mono‐energetic images (VMI) spectral result mean HU values of the eight material rods as a function of phantom/patient size. Scans were performed with the nominal pediatric abdomen protocol (100 mAs). The 67 keV VMI result was found to present the closest size‐averaged HU values to those of the conventional result. Left: quantification results. Right: differences in the quantification results from the size‐averaged HU value. Similar to the 70 keV VMI spectral results, a significantly increased size stability (independence) is observed for the 67 keV VMI result, as compared to the conventional result.

Similarly, our analysis includes a size dependency of an additional set of VMI results, whose energy level (67 keV) was selected based on their closest quantification values to those of the conventional result. Figure 3 presents the size dependency of the conventional images compared to that of the 67 keV VMI spectral results. Here too, the vertical trends that appear in Fig. 3(a) are emphasized in Fig. 3(b), which shows measured values relative to the average quantification of each material per each result. While the same observations that were made for the 70 keV VMI hold also to for the 67 keV VMI, a slight increase in the maximal absolute difference of the HA‐400 material rod (13.8 HU) indicates a reduction of the size dependent consistency improvement factor from 3.6 to 2.9. Bland‐Altman analysis for the 67 keV VMI spectral result reveals 95% “limits of agreement” of −2.72 ± 1.01 HU (10 cm vs 15 cm patient size) and −5.54 ± 2.84 HU (15 cm vs 20 cm patient size).

**FIG. 4:**
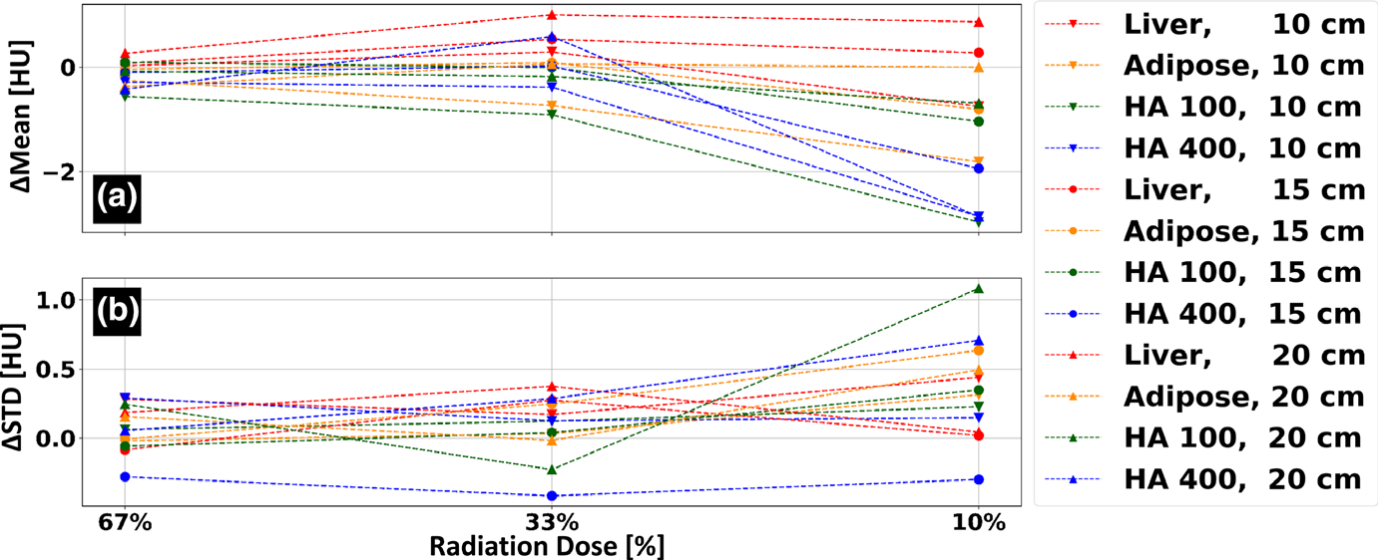
Dose dependency results for the 70 keV virtual mono‐energetic images (VMI) results. (a) Average HU values relative to those measured using the clinical protocol at 67%, 33% and 10% radiation dose of the clinical protocol (9 mGy, 100 mAs at 120 kVp). (b) Corresponding change in standard deviation (STD) relative to the standard deviation values measured using the clinical protocol.

Figure 4 presents the radiation dose dependency of the 70 keV VMI spectral result. Relative to the nominal (100 mAs, 9 mGy) acquisitions, mean HU quantifications are accurate within a single HU for 33% and 67% dose reduction acquisitions, and within −3 to 1 HU for the 90% dose reduction acquisitions [Fig. 4(a)]. Changes within ±0.5 HU for the 33% and 67% dose reduction acquisitions and within a single HU for the 90% dose reduction acquisitions of the standard deviation between measurements were observed (except for the HA‐100 rod, when acquired at 10% of the nominal dose within the largest phantom size configuration).

**FIG. 5:**
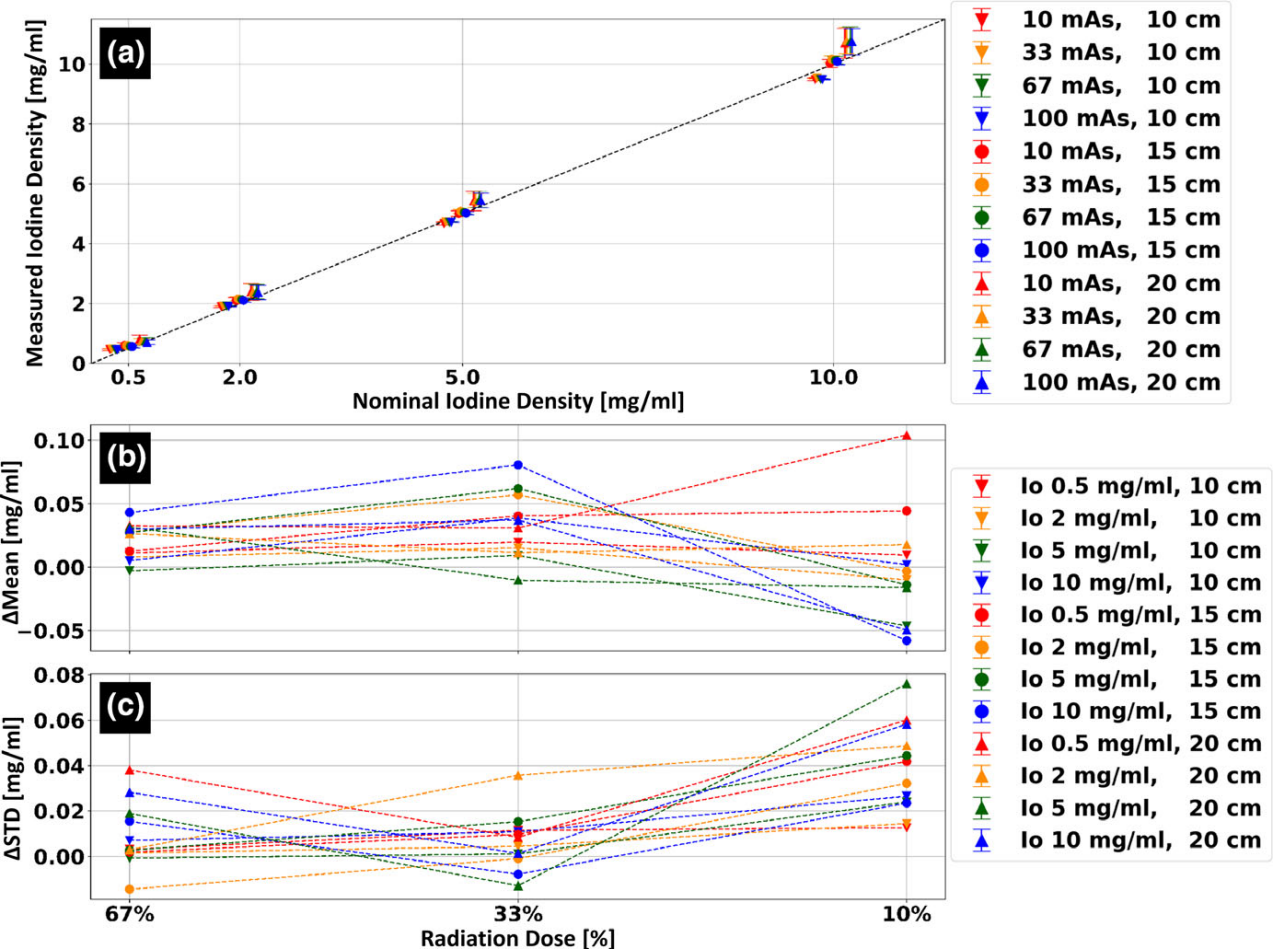
Size and radiation dose stability of the iodine density spectral result. (a) Average iodine density values, in [mg/ml], for four different iodine concentrations. Scans were performed for three different phantom/patient sizes and at four different dose levels). (b) Average iodine concentration values, relative to those measured using the clinical protocol, at 67%, 33% and 10% radiation dose of the clinical protocol (9 mGy, 100 mAs at 120 kVp). (c) Corresponding change in standard deviation (STD) relative to the standard deviation values measured using the clinical protocol.

Figure 5 presents iodine quantification stability as a function of patient size and radiation dose. Patient size stability between the different patient sizes was quantified with standard deviations of 0.1, 0.2, 0.3, and 0.5 mg/ml for the 0.5, 2, 5, and 10 mg/ml rods, respectively. For all iodine densities, measurements of iodine quantification dependency on radiation dose revealed mean iodine density changes within 0.04 mg/ml when applying 67% of the nominal radiation exposure, within 0.08 mg/ml when applying 33% of the nominal radiation exposure, and within 0.1 mg/ml when applying 10% of the nominal radiation exposure. Changes in standard deviation relative to those measured for the nominal radiation exposure were within ±0.08 mg/ml for all reduced dose acquisitions.

**FIG. 6:**
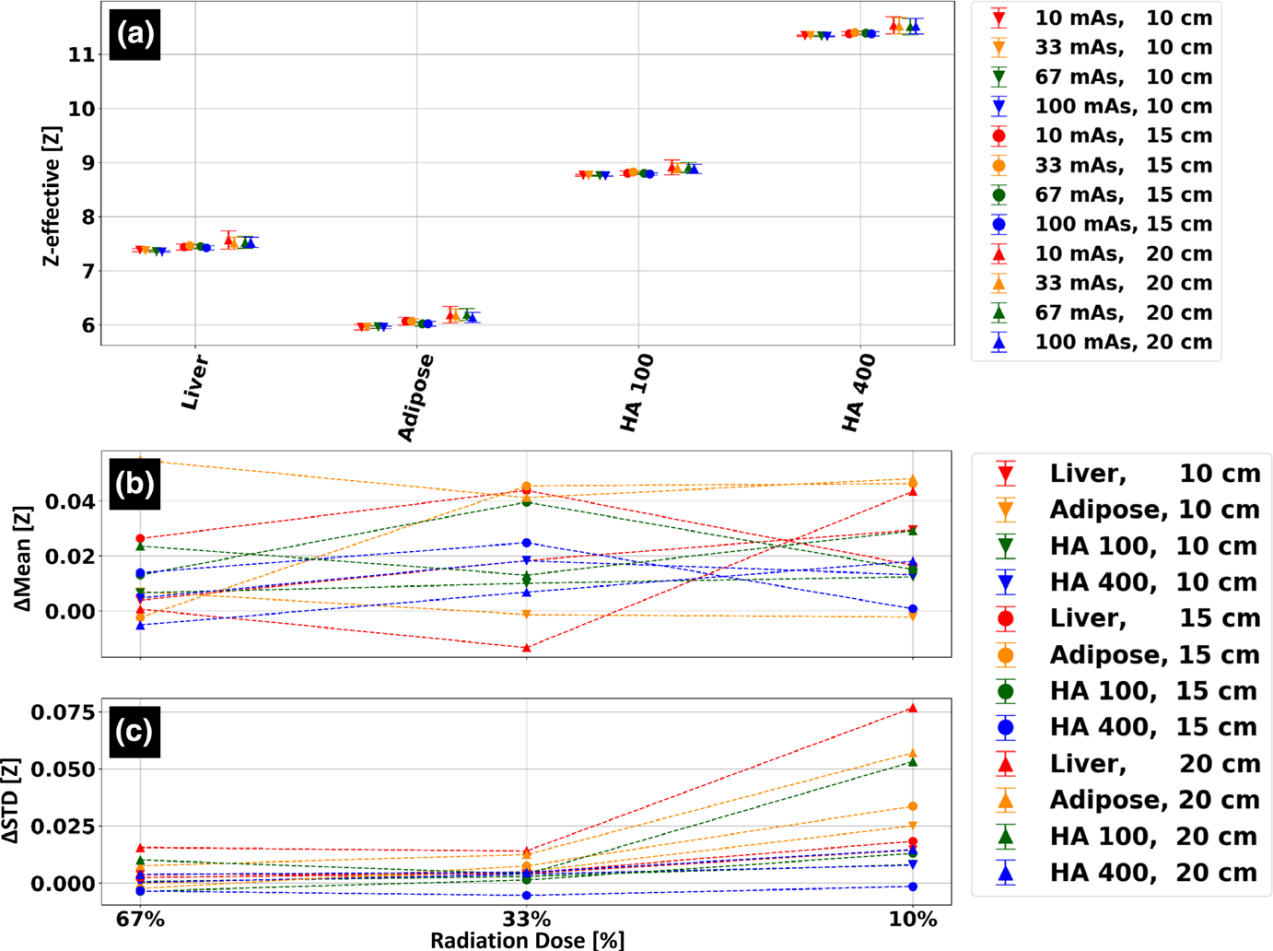
Size and radiation dose stability of the Z‐effective spectral result. (a) Average effective atomic number values for four different tissue‐mimicking materials. Scans were performed for three different phantom/patient sizes and at four different dose levels). (b) Average effective atomic number values, relative to those measured using the clinical protocol, at 67%, 33% and 10% radiation dose of the clinical protocol (9 mGy, 100 mAs at 120 kVp). (c) Corresponding change in standard deviation (STD) relative to the standard deviation values measured using the clinical protocol.

Figure 6 presents effective atomic number (Zeff) stability as a function of patient size and radiation dose. Zeff for all tissue‐mimicking materials demonstrated relatively low dependence on patient size. Maximal differences of 0.19, 0.23, 0.15, and 0.19 atomic numbers were measured for the liver, adipose, HA‐100 and HA‐400 materials, respectively. The dependency of Zeff quantifications on radiation dose was observed as a relative change of up to 0.06 atomic numbers and an increase of 0.08 in standard deviation for all material inserts at all phantom sizes and all radiation dose levels.

**FIG. 7:**
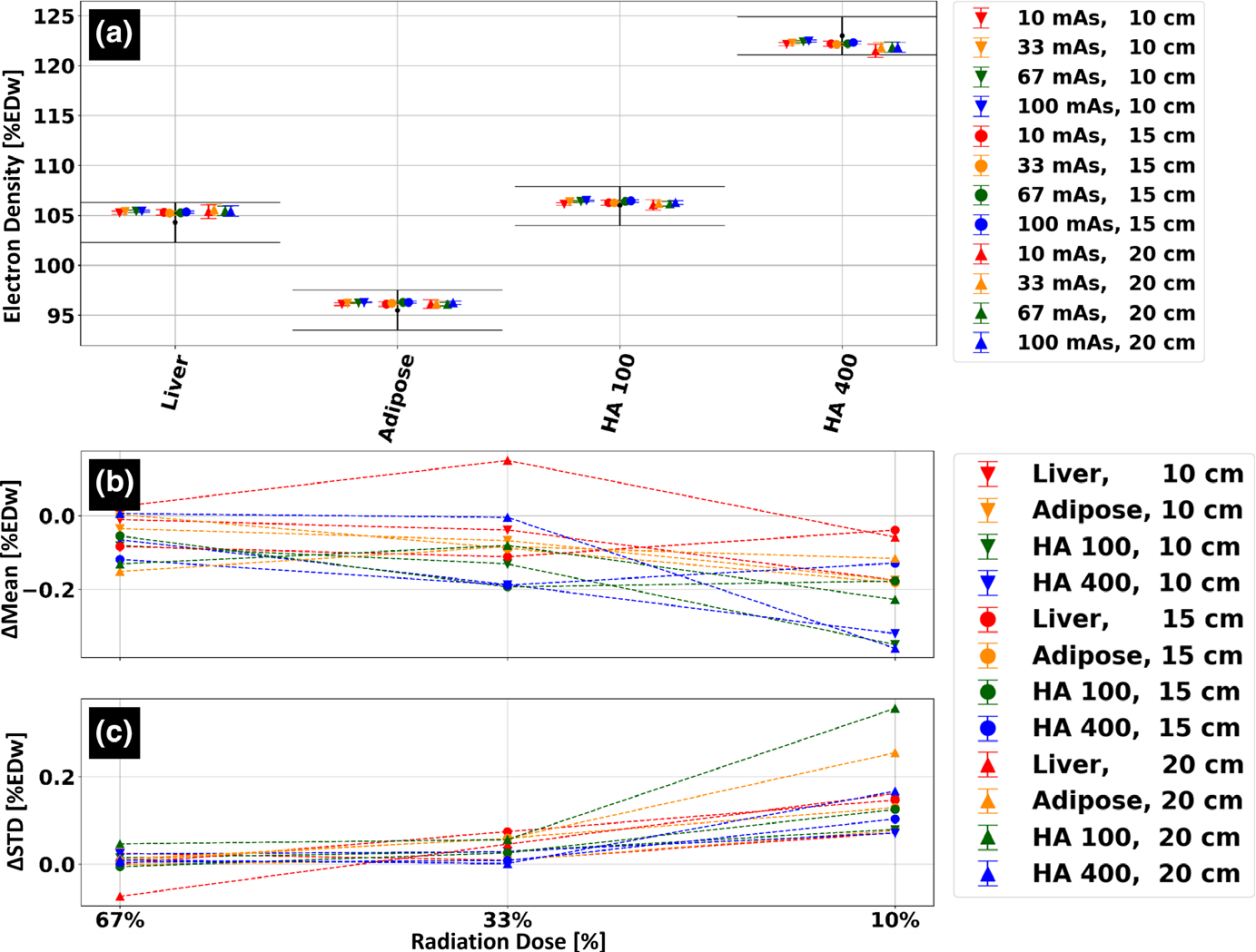
Size and radiation dose stability of the electron density spectral result. (a) Average electron density values, in [%ED_water_], for four different tissue‐mimicking materials. Scans were performed for three different phantom/patient sizes and at four different dose levels. Black error bars indicate the nominal value for each material rod including deviations due to physical density uncertainties. (b) Average electron density values, relative to those measured using the clinical protocol, at 67%, 33% and 10% radiation dose of the clinical protocol (9 mGy, 100 mAs at 120 kVp). (c) Corresponding change in standard deviation (STD) relative to the standard deviation values measured using the clinical protocol.

Electron density (ED) accuracy and stability as a function of patient size and radiation dose is presented in Fig. 7. Relative to the uncertainty of the nominal ED values due to density variations of the material rods [black error bars, Fig. 7(a)], all mean measured values are accurately quantified. Stability of ED estimations between different patient sizes was quantified with standard deviation of 0.35, 0.2, 0.26, and 0.71 %ED_water_ for the liver, adipose, HA‐100 and HA‐400, respectively. For all tissue‐mimicking materials, measurements of the ED estimation dependency on radiation dose revealed mean values within 0.2 %ED_water_ when applying 67% and 33% of the nominal radiation exposure, and within 0.36 %ED_water_ when applying 10% of the nominal radiation exposure. Changes in standard deviation relative to those measured for the nominal radiation exposure were within ±0.1 %ED_water_ for the 67% and 33% reduced dose acquisitions and up to 0.32 %ED_water_ for the 10% reduced dose acquisitions.

**FIG. 8:**
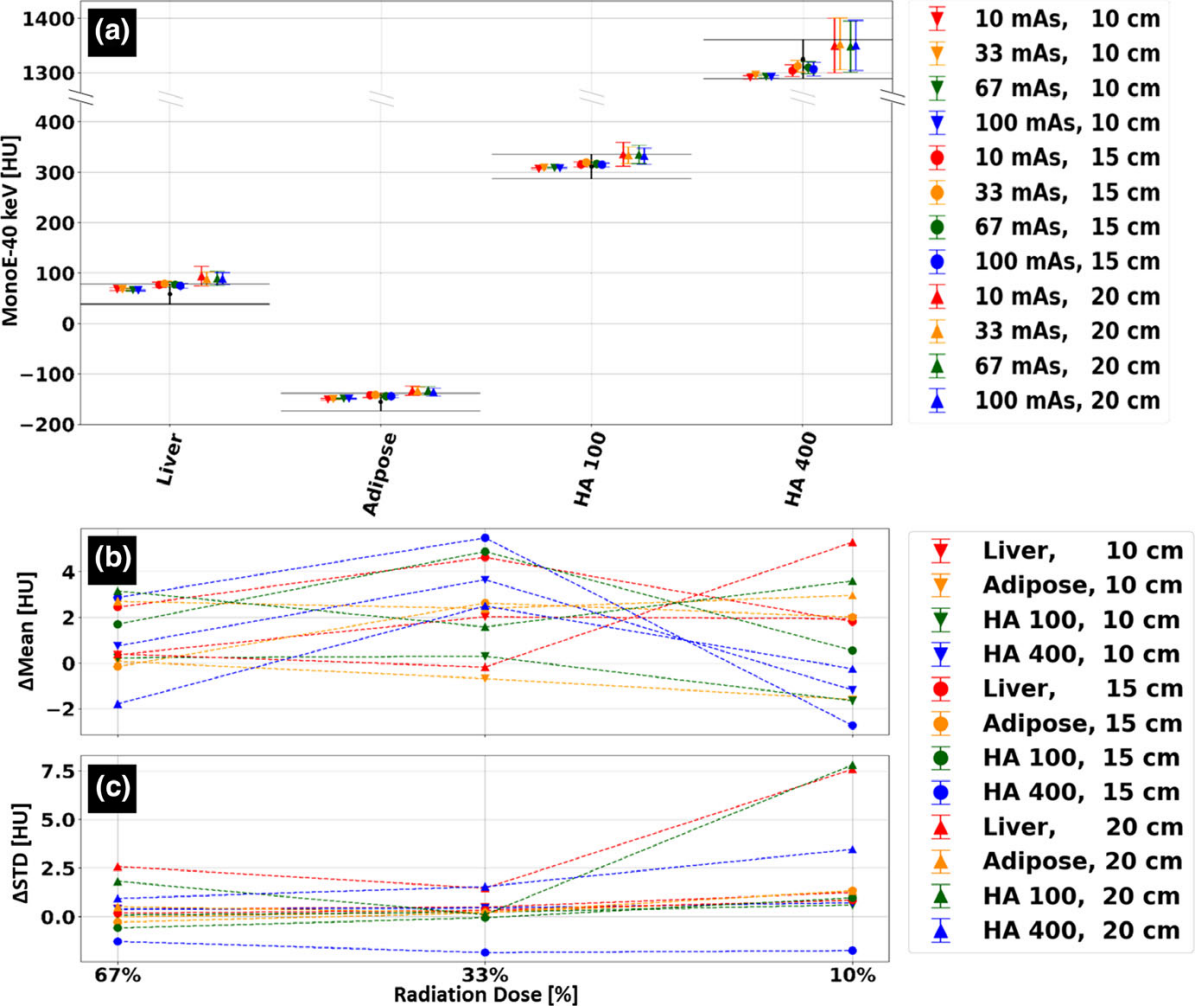
Size and radiation dose stability of the 40 keV virtual mono‐energetic images (VMI) spectral result. (a) Average 40 keV VMI values, in [HU], for four different tissue‐mimicking materials. Scans were performed for three different phantom/patient sizes and at four different dose levels. Black error bars indicate the nominal value for each material rod including deviations due to physical density uncertainties. (b) Average HU values, relative to those measured using the clinical protocol, at 67%, 33% and 10% radiation dose of the clinical protocol (9 mGy, 100 mAs at 120 kVp). (c) Corresponding change in standard deviation (STD) relative to the standard deviation values measured using the clinical protocol.

VMI attenuation quantification accuracy was evaluated for the additional keV energies 40, 50, 60, 100, 150, and 200 keV. Due to the increased values of attenuation at lower energies, observed patient size dependencies and radiation dose dependencies were largest for to 40 keV VMI (Fig. 8) and decreased for higher VMI energies. Size dependency quantifications, calculated as the maximal difference in mean HU across all radiation dose levels for each of tissue‐mimicking materials, and radiation dose dependency quantifications, calculated as the maximal deviation of mean HU from the nominal radiation exposure across all patient sizes and tissue‐mimicking materials, is presented in Table 2. Except for the lowest energy level of 40 keV, all VMI images demonstrated radiation dose dependency below 2 HU when applying down to 33% of the nominal radiation exposure and below 3.7 HU when applying down to 10% of the nominal radiation exposure.

**TABLE 2:** Patient size dependency, calculated as the maximal quantification difference between different phantom sizes, and radiation dose dependency, calculated as the maximal quantification difference relative to the nominal (100 mAs, 9 mGy) dose level, for different virtual mono‐energetic images (VMI) results. All values are given in HU.

	Max. patient size dependency [HU] (across all dose levels)				Max. dose dependency [HU] (across materials/sizes)		
	Liver	Adipose	HA‐100	HA‐400	67 mAs	33 mAs	10 mAs
40 keV	26.1	17.4	28.8	58.7	3.2	5.5	5.3
50 keV	16.8	11.1	17.8	34.5	1.5	2.7	3.0
60 keV	11.4	7.3	11.4	20.6	0.7	1.3	2.6
70 keV	8.1	5.2	7.6	12.2	0.6	1.0	3.0
100 keV	4.3	2.3	2.5	2.0	1.1	1.3	3.4
150 keV	3.9	1.6	1.8	5.3	1.4	1.8	3.7
200 keV	3.6	2.0	2.3	6.5	1.6	2.0	3.7

## 4 DISCUSSION

Quantitative medical imaging is receiving greater acknowledgment from clinicians and healthcare providers due to its rapidly increasing clinical value.^34^, ^35^ Utilization of spectral CT applications for pediatric patients is still in its early adoption stages, with a pressing lack of quantitative evaluations to determine the spectral capabilities for this unique patient population. The dose‐neutrality of dual‐energy CT, compared to conventional CT,^23^, ^36^ provides an opportunity to improve patient care also for the pediatric population. For example, the use of quantitative measurements could be very helpful for lesion characterization^37^ and in the analysis of tumor treatment response.^38^ Proton stopping power in proton therapy is another area of research.^39^

In our study, we evaluated quantification accuracy and stability with respect to patient size and radiation dose of spectral results from a spectral detector CT scanner. Our analysis of various material measures, such as virtual mono‐energetic attenuation, effective atomic number, and electron density, revealed that available spectral results from a spectral detector‐based CT system provide high material quantification stability across the three different pediatric patient sizes tested.

Compared to the conventional result, VMI images at energies 70 and 67 keV provide better consistency between different pediatric patient sizes, where attenuation values provided by the 67 keV VMI result were closest to those provided by the (120 kVp) conventional images.

A high level of spectral accuracy was achieved with the highest radiation dose and reference patient size chosen for this study. For all measured VMI energies, spectral quantifications were within ±1.5% of their nominal CT numbers. In addition, in the keV range of 60 to 200 keV, deviations from nominal values of soft tissue‐mimicking materials (Liver, Adipose and HA‐100) were within 3 HU. ED quantifications were within ±1.0%, and iodine density and Zeff values were within 0.1 mg/ml and 0.26 atomic numbers of their nominal values, respectively.

Spectral results were found to have good consistency with respect to patient size, as measured for three different (pediatric) patient sized phantoms. Maximal differences in measured attenuation below 2.5%, 1.8% and 1.2% for the 40, 50 and 60 keV VMIs and below 0.8% for the rest of the keV energies attest to the strong ability of spectral CT imaging to provide consistent attenuation quantifications for different patient populations. Such an improvement in consistency, compared to that of conventional CT images (Figs. 2 and 3), is mainly due to correct accounting of beam‐hardening effects and should allow the development of quantitative differential measures that could be applied for a large range of (early) body developmental stages. Lesion characterization, tumor treatment response evaluations and renal calculi composition assessments, for example, will greatly benefit from consistent HU values between different patient sizes.

Most importantly, the significant robustness of spectral quantification with respect to radiation dose is extremely important with respect to the long‐standing debate on radiation exposure for children.^20^, ^40^ Excluding the lowest VMI energy of 40 keV, and relative to the nominal clinical protocol, quantification accuracy of all tissue‐mimicking materials was maintained below 1.6 HU for a 33% dose reduction, below 2.7 HU for a 67% dose reduction and below 3.7 HU for a dose reduction of 90%. At the 90% dose reduction level, iodine, ED, and Zeff quantifications were stable to within 0.1 mg/ml, 0.36 %ED_water_ and 0.06 atomic numbers, respectively. This robustness implies that the high accuracy that was quantified with the highest radiation dose is almost completely maintained at reduced radiation dose levels. High spectral quantification accuracy enables improved diagnostic capabilities and confidence for a range of clinical applications.^3^, ^4^, ^9^, ^37^, ^38^ While shown to be dose‐neutral in the past,^23^, ^36^ the ability to provide accurate spectral results at the dose reduction levels demonstrated in our study enables radiologists to further utilize these capabilities without compromising the safety of pediatric patients.

Our study did have limitations. Primarily, our experiment only included evaluations of the spectral performance from a single type of spectral CT technology. Other clinically available DECT implementations, such as dual‐source or fast kVp‐switching technologies, should also be evaluated in future studies in order to allow for a consensus on the general applicability of spectral CT for pediatric evaluations. In addition, while our study included four different radiation dose levels, i.e., 100%, 67%, 33% and 10% of the nominal level, it included only a single examination protocol. However, according to theoretical background of spectral detector CT imaging and to previous studies,^6^, ^13^ the performance of the spectral detector CT solution should be independent of acquisition parameters such as rotation time, collimation, and pitch. This is due to the simultaneous acquisition of signals from the two spectra, which is unique to the detector‐based DECT approach enables. Notably, the pediatric protocol that we utilized in this study was already configured with the fastest rotation time available on the system (0.27 s per rotation), extremely important for the pediatric population. Finally, our study included only phantoms and did not involve any clinical data. Moreover, the phantoms that were used in our study were not anthropomorphic phantoms but rather a material quantification phantom, designed for spectral quantification evaluation purposes and equipped with two extension rings that simulate different pediatric patient sizes. The authors are unaware of commercially available pediatric‐sized anthropomorphic phantoms that are adequate for evaluating DECT material quantifications. For our purposes of quantifying the accuracy, consistency and stability of spectral results, a phantom study is the only viable solution for material quantifications with known ground truths and for performing dose dependency evaluations without introducing risk to a vulnerable patient population. However, while phantoms are important as an initial evaluation step, the benefits of the stability and consistencies of spectral results will require validation and further assessments in dedicated clinical studies and with respect to specific clinical applications for pediatric patients.

## 5 CONCLUSION

Spectral results from a spectral detector DECT scanner provide accurate and consistent material characterizations with respect to different pediatric patient sizes and radiation dose reduction of up to 90%. The stability of the material quantifications such as mono‐energetic attenuation estimations, as well as iodine density, effective atomic number and electron density quantifications enables an important step towards quantitative CT imaging for children of various ages with low exposure to ionizing radiation.

## AUTHOR CONTRIBUTION STATEMENT

NS performed the measurements and participated in the analysis and in writing the manuscript, KM participated in the analysis and in writing the manuscript, PBN participated in the analysis and in writing the manuscript.

## CONFLICTS OF INTEREST

The authors declare no conflict of interest.
